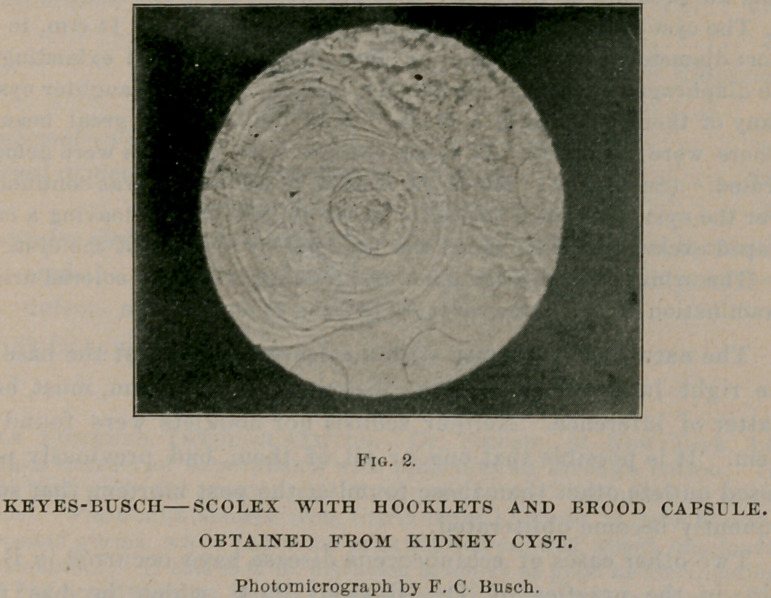# A Case of Multiple Echinococcus Cysts

**Published:** 1896-08

**Authors:** William C. Keyes, Frederick C. Busch

**Affiliations:** Laboratory of Pathology, University of Buffalo, 24 High Street; Laboratory of Pathology, University of Buffalo, 24 High Street


### A CASE OF MULTIPLE ECHINOCOCCUS CYSTS.

By WILLIAM C. KEYES, M. D., and FREDERICK C. BUSCH, B. S.,
Laboratory of Pathology, University of Buffalo.

MR. S., aged 53, German. His father died at the age of 71 ; his mother is living, aged 80. He was the last of four children. His habits were extremely intemperate. He often slept out of doors nights. He had typhoid fever in 1865 and some liver trouble in 1872. At this time, according to his statement, he passed a quantity of black marbles, probably gall stones.

His last illness dates from January, 1895. March 17, 1895, he came under the care of Drs. Hebenstreit and Meisburger. The clinical history, as obtained from them, is as follows :

He had pain in the right side, in the neighborhood of the liver ; he coughed up greenish fluid, of a disagreeable odor and an acrid taste. The right chest was bulging; the right pleural cavity was aspirated and one syringeful of serous fluid removed. Small tumor-like masses, which could be felt just below the ensiform cartilage and to the right, were also explored with the hypodermic needle, with no result. Upon percussion the liver flatness did not extend downward below the free border of the ribs; it extended as high as the nipple in front and, at the same level, to the median line behind. In the axillary line there was flatness, extending to the crest of the ilium. There was an inguinal hernia on the left side.

The patient died March 16, 1896. The post mortem was performed thirty-six hours after death. The subject was emaciated. Rigor mortis was firm. There was the usual amount of post mortem discoloration of dependent parts.

*Heart.*—The pericardium was empty; the heart was of normal size; the muscle was flabby, soft and fatty degenerated; the leaflets of the aortic valves were calcareous at the base; the other valves were normal; the aorta appeared normal.

*Lungs.*—There was no fluid in the pleural cavities; the left lung crepitated well and was normal. The right lung was firmly adherent at the base to the diaphragm; it crepitated well. On section, bloody froth exuded from the bronchial tubes. A cavity with calcareous walls was discovered in the base of the lower lobe, measuring 50 mm. in breadth and 10 mm. in depth. It communicated with a bronchus on the one hand and on the other, through an opening in the diaphragm, with a similar though much larger cavity, immediately below the diaphragm. The latter, which was intimately connected with the diaphragm, measured 100 mm. in its horizontal diameter and 15 mm. in depth. It was also lined by calcareous plates. No opening could be discovered leading from it excepting that into the lung cavity.

Immediately beneath this subdiaphragmatic cavity was still another. It was partially surrounded by liver tissue and was intimately connected to the gall bladder and its duct by inflammatory adhesions. Its capacity was about 100 c.c. No connection could be demonstrated between it and the cavity above or with the gall bladder and ducts. The calcareous lining of all three cavities was bile stained.

The peritoneal cavity contained about 1,500 c.c. of serous fluid. In the omentum and mesentery were ten or twelve nodules, varying in size from that of a pea to a walnut and soft in consistence. On the left side, attached to the sac of the inguinal hernia, was one of these nodules. On section they were found to consist of concentric layers of gelatinous substance. Some of them showed cheesy and calcareous material. On microscopical examination hooklets were demonstrated in them.

The liver was small. The left lobe was large, somewhat distorted

FIG. 1.

KEYES-BUSCH—RIGHT KIDNEY WITH CYST. DORSAL VIEW. X. 5.

From a photograph by F. C. Busch.

FIG. 2.

KEYES-BUSCH—SCOLEX WITH HOOKLETS AND BROOD CAPSULE. OBTAINED FROM KIDNEY CYST.

Photomicrograph by F. C. Busch.

and displaced by the pressure of a large cyst connected with the right kidney. The right lobe was greatly diminished in size, probably owing to the pressure of the cyst. Microscopically the liver showed much pigmentation, a large increase in connective tissue and some atrophy of epithelial cells.

The gall bladder contained about 15 c.c. of bile. It was normal in size ; its mucous membrane appeared normal. Its duct was about the size of a quill. The hepatic duct contained a stone, oval in shape and about 1.5 c.m. in diameter.

The spleen was normal. The pancreas was fatty and showed an increase of connective tissue.

The stomach was somewhat dilated. The mucosa showed large dilated venules and ecchymoses and was covered by shining mucus. It was also much pigmented.

*Intestines.*—The lower part of the ilium was edematous, thick and greenish in hue. Upon section the thickening was seen to be chiefly of the peritoneal coat. There was also an increase of connective tissue in the submucosa. Several other parts of the small intestine showed limited areas of hemorrhagic infiltration, but no appreciable thickening. The appendix was entirely covered by peritoneum. It was about 10 c.m. in length and attached by adhesions to the brim of the pelvis.

The left kidney was large and pale. The right kidney was covered anteriorly by a large cyst. The anterior and upper half of this kidney was atrophied to half its normal size ; the posterior half was flattened from the pressure of the cyst. (See Fig. 1.)

The cyst measured 18 c.m. in its long diameter and 14 c.m. in its short diameter, pressing upon the right kidney below and extending to the diaphragm above. It contained a large number of daughter cysts, many of them filled with a clear, limpid fluid, and of great beauty. Others were collapsed. In these, scolices with hooklets were demonstrated. (See Fig. 2.) Since the capsule of the kidney was continuous over the cyst, the latter probably arose in the cortex, leaving a cup-shaped excavation in it, which was occupied by one end of the cyst.

The urinary bladder contained about 47 c.c. of amber-colored urine. Examination of the latter revealed nothing of importance.

The nature of the cavity with a calcareous lining at the base of the right lung and of the two below the diaphragm, must be a matter of inference. Neither scolisis nor hooklets were found in them. It is possible that one or all of them had previously possessed outlets other than those found at the post mortem, that subsequently became obliterated.

Two other cases of echinococcus disease have occurred in Buffalo, in the practice of Dr. Roswell Park, which he has not yet reported.

The disease is exceedingly rare in this country, Osler having been able to collect only eighty-five cases in the United States and Canada. Of these there were but three in which the cysts occurred in the kidney.

24 HIGH STREET.

## Figures and Tables

**Fig. 1. f1:**
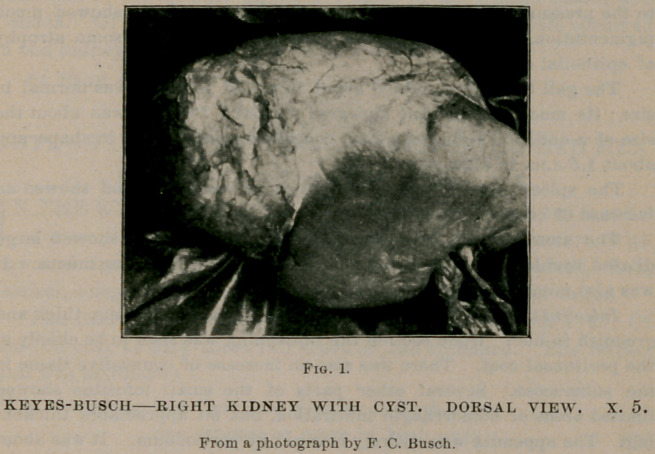


**Fig. 2. f2:**